# Effects of *COLQ* Gene Missense Mutations on Growth and Meat Traits in Leizhou Black Goats

**DOI:** 10.3390/ani15172618

**Published:** 2025-09-06

**Authors:** Jing Huang, Ke Wang, Yuelang Zhang, Jiancheng Han, Hanlin Zhou, Qinyang Jiang

**Affiliations:** 1 Guangxi Key Laboratory of Animal Breeding, Disease Control and Prevention, College of Animal Science and Technology, Guangxi University, Nanning 530004, China; huangjing07182024@163.com; 2 Zhanjiang Experimental Station, Chinese Academy of Tropical Agricultural Sciences, Zhanjiang 524013, China; lp_wangke@163.com (K.W.); hanjiancheng810@163.com (J.H.); 3 Sanya Research Institute, Chinese Academy of Tropical Agricultural Sciences, Sanya 572024, China; 4 Hainan Institute of Zhejiang University, Sanya 572024, China; zhangyuelang@zju.edu.cn

**Keywords:** *COLQ*, genetic variants, goat, productivity performance, association analysis

## Abstract

As an indigenous goat breed unique to southern China, Leizhou Black Goats (LZBGs) are highly valued for their rapid growth, high reproductive performance, and superior meat quality. However, their offspring frequently exhibit symptoms of muscle atrophy and malnutrition, suggesting potential genetic defects underlying these adverse phenotypes. As a unique extracellular matrix component, collagen Q (*COLQ*) is specifically enriched within the synaptic basal lamina at vertebrate neuromuscular junctions (NMJs), where it anchors acetylcholinesterase (AChE) to facilitate efficient acetylcholine hydrolysis, ensuring precise neuromuscular signaling. The current investigation sought to characterize the spectrum of genetic polymorphisms within the *COLQ* gene and assess their correlation with key production traits, including growth performance and meat quality parameters, in the LZBG population. Previously, through whole-genome sequencing and transcriptome sequencing analyses of an LZBG population, we identified four SNPs in the *COLQ* gene, namely, two missense mutations (SNP1: p.238A/S and SNP3: p.47G/S), one intronic variant (SNP2), and one synonymous mutation (SNP4: p.101P/P). Population genetic analysis revealed strong linkage disequilibrium between SNP1 and SNP2. Computational modeling of protein structures predicted that the identified missense mutations may lead to alterations in protein conformation. Association analyses demonstrated significant correlations of SNP1 and SNP3 with growth and meat quality traits (*p* < 0.05), where SNP3 reduced *COLQ* expression by 0.64-fold in homozygotes. Association analysis revealed that both SNP1 and SNP3 showed significant correlations with growth and meat quality traits in LZBGs (*p* < 0.05). Notably, SNP3 (p.47G/S) was found to regulate *COLQ* gene expression, reducing its levels by 0.64-fold in homozygous individuals, suggesting its potential as a genetic marker for selecting goats with superior growth performance and muscular development characteristics. The identified genetic variants establish a foundation for marker-assisted selection in LZBG breeding programs with particular relevance to growth performance enhancement, while also advancing the understanding of *COLQ*’s functional mechanisms in muscle development.

## Simple Summary

The Leizhou Black Goat is a prized local breed from southern China, known for growing quickly and providing high-quality meat. However, many young goats suffer from poor growth and weak muscles, which is a problem for farmers. We suspected that this might be caused by tiny, natural changes in their genes. Our study focused on one specific gene called *COLQ*, which is important for muscle function. We discovered several natural variations within this gene. Two of these variations were found to be directly linked to the goats’ size and meat quality. One variation, in particular, significantly reduces the activity of the *COLQ* gene. This discovery is very useful because it allows breeders to use a simple genetic test to select parent goats that are more likely to produce healthy, strong offspring with better growth. This will help improve the sustainability and productivity of goat farming.

## 1. Introduction

Goat husbandry, concentrated in tropical and semi-arid regions of developing countries, serves as a key income source for smallholders within mixed farming systems [1]. Compared to cattle and monogastric livestock, goats offer higher economic viability due to lower costs, faster reproduction, diversified products (meat, milk, and fiber), efficient use of byproducts, and strong resilience to drought and heat stress [2]. Furthermore, goat meat provides a nutritious red meat option with low cholesterol and fat content [3].

The Leizhou Black Goat (LZBG), an indigenous ruminant breed native to tropical China, demonstrates remarkable thermo-hygric adaptability, coupled with superior production traits, including accelerated growth kinetics, enhanced reproductive performance, and premium meat quality characteristics [4,5]. However, this breed frequently exhibits juvenile myopathic manifestations and nutritional deficiencies, even under conditions of adequate milk supply and proper nutrition, suggesting an underlying hereditary myopathy. These congenital conditions, often attributed to genetic mutations affecting neuromuscular signal transmission [6], muscle protein structure [7], or cellular metabolism [8], ultimately compromise production efficiency and commercial viability [9]. Therefore, screening for candidate genes and genetic variations related to growth and muscle development is of great significance for the breeding of LZBGs.

Collagen Q (COLQ), a specialized non-fibrillar collagen, plays an essential role in organizing the vertebrate neuromuscular junction by anchoring synaptic acetylcholinesterase (AChE) and participates in MuSK-dependent signaling for synaptic (CMS) regulation [10]. CMS are a group of inherited disorders caused by genetic mutations in the neuromuscular junction (NMJ), characterized by activity-dependent muscle weakness and deficient AChE function. Mutations in COLQ are known to cause congenital myasthenic syndromes across species [11]. For instance, Rinz et al. identified a pathogenic *COLQ* variant (I337T) in Labrador Retrievers leading to generalized myopathy [12], while Abitbol et al. reported a homozygous missense mutation in Sphinx and Devon Rex cats resulting in progressive muscle weakness due to disrupted AChE aggregation [13]. Laforgia et al. documented a case involving a large homozygous deletion in the *COLQ* C-terminal domain [14]. More recently, Ncube et al. associated the *COLQ* gene with carcass fat content and muscle content, suggesting a broader influence on muscle traits [15]. Once the CMS caused by such genetic mutations is inherited and accumulates within the population, it will lead to a sharp decline in both the quality and yield of livestock products. Fortunately, this genetic disorder can be efficiently eliminated through early screening. However, as of now, there are fewer reports related to *COLQ* gene in goats. Similar to these studies, our previous analysis in goats screened out *COLQ* too [16]. Given this context, we hypothesize that genetic variations in the *COLQ* gene regulate muscle development in goats, thereby influencing growth and meat quality traits.

This study sought to characterize *COLQ* gene expression profiles and identify potential SNPs within *COLQ* in LZBGs, investigating their associations with growth and muscle development. Additionally, correlations between *COLQ* genotypes and mRNA expression levels were assessed. These findings may serve as a valuable reference for goat breeding programs, advance functional studies of the *COLQ* gene, and uncover potential molecular markers for MAS applications.

## 2. Materials and Methods

### 2.1. Feeding Environment and Management

This study utilized experimental animals housed at the Leizhou Black Goat Farm in Danzhou, Hainan Province. All animals were maintained on an identical feeding regimen and raised under standardized conditions [17]. All experimental protocols received approval from the Animal Care and Use Committee of the Chinese Academy of Tropical Agricultural Sciences (CATAS-2025002ZES), with all procedures, including sample collection, conducted in strict compliance with institutional ethical guidelines (GB/T 35892-2018) [18].

### 2.2. Sample and Phenotypic Data Collection

Ear tissue samples were randomly collected from healthy, multiparous (having given birth multiple times) female Leizhou Black Goats, all approximately 2 years of age. Growth-related traits, including CC (chest circumference), CW (chest width), BL (body length), CD (Chest depth), BH (body height), WH (withers height), HW (hip width), and BW (body weight), were systematically measured and documented in all 1010 experimental animals. A subset of 98 two-year-old animals was randomly selected from the population for carcass evaluation, including measurements of water loss rate (WLR), shear force (SF), carcass weight (CW), cross-section area of longissimus dorsi lumbois muscle (CALM), and water holding capacity (WHC). The specific collection method strictly follows the Chinese agricultural industry standard NY/T 630-2002 [19]. For comprehensive gene expression analysis, multiple tissue types (including skin, heart, gluteofemoral biceps, longissimus dorsi muscle, rumen, liver, kidney, abdominal adipose, cerebrum, and cerebellum) were harvested from 18 randomly selected adult female animals. Longissimus dorsi muscle specimens (N = 5 × 6 = 30) were obtained from LZBGs at defined developmental timepoints, namely, 0 days (neonatal), 6 months, and 1, 2, 3, and 4 years of age, enabling developmental expression profiling. Concurrently, longissimus dorsi muscle samples (N = 32) from 2-year-old females were analyzed to determine genotype-dependent effects of missense mutations on *COLQ* transcript levels. After the slaughtering and cutting of all the tissue samples, except for the fresh samples required for meat quality testing, they were immediately snap-frozen in liquid nitrogen and subsequently stored at −80 °C for long-term preservation.

### 2.3. Genomic DNA and Total RNA Extraction

Genomic DNA extraction from ear tissue samples was performed with the Animal Tissues/Cells Genomic DNA Extraction Kit (Solarbio, Beijing, China), followed by purity and concentration assessment using a NanoDrop™ 1000 Spectrophotometer (Thermo Fisher Scientific, Waltham, MA, USA). The DNA samples were diluted to 20 ng/μL using ddH_2_O and stored at −20 °C until further analysis. Total RNA was extracted using the TRIzol method (Solarbio, Beijing, China), and the first-strand cDNA was synthesized by PrimeScript™ RT Reagent Kit (Takara, Tokyo, Japan) following the manufacturer’s protocol.

### 2.4. Primer Synthesis, Amplifications, and Genotyping

Based on the GGVD (Goat Genome Variation Database, http://animal.omics.pro/code/index.php/GoatVar, accessed on 20 July 2024), 18 LZBGs’ whole genome sequencing (WGS) and RNA-seq results (Supplementary Materials, File S1), 2 SNPs (chr1:152339819C>A; chr1:152348368C>T) on the LZBG *COLQ* gene were identified [16]. Subsequently, using the caprine *COLQ* gene reference sequence (NC_030808.1) as a template, four specific primer pairs were designed using the NCBI Primer-BLAST tool (last accessed: 1 August 2024) to amplify target regions. Exon-spanning primers were designed to quantify *COLQ* mRNA expression levels, using *GAPDH* as the endogenous control (Table S1). Conventional and quantitative PCR amplifications were performed following established protocols (Chen et al., 2015) [20]. *COLQ* variants were subsequently identified through Sanger sequencing. SNPs were detected via sequence alignment against the reference genome using BioXM software (v2.7.1; Nanjing Agricultural University, China).

### 2.5. Statistical Analysis

The analysis was performed using the Wei Sheng Xin program (https://www.bioinformatics.com.cn/, accessed on 8 May 2025) and GenAlEx (accessed on 12 May 2025) to calculate the linkage disequilibrium (LD) structure, population genetic parameters, Hardy–Weinberg equilibrium (HWE), and polymorphic information content (PIC) of SNP loci in the goat *COLQ* gene. Phylogenetic conservation and evolutionary relationships of *COLQ* across species were analyzed using Ensembl’s gene tree and ortholog prediction tools. The COLQ protein structure was predicted via homology modeling using Swiss-Model (accessed 29 May 2025). Associations between *COLQ* SNPs and growth/muscle traits in Leizhou Goats were evaluated via the general linear model [9,21] (SPSS v23.0; IBM, Armonk, NY, USA), with the following model:

Y_sj_ = μ + G_s_ + e_j_

where Y_sj_ represents the phenotypic value, μ denotes the population mean, G_s_ indicates the genotype effect, and e_j_ signifies random error. Relative *COLQ* expression levels were calculated using the 2^−ΔΔCt^ method [22], and the data derived from qPCR were analyzed using one-way analysis of variance followed by a post-hoc test. All of the data were presented as the mean ± standard error (S.E.), and *p* < 0.05 was considered to be significant.

## 3. Results

### 3.1. Identification of Missense Mutations in Goat COLQ Gene

**Figure 1:**
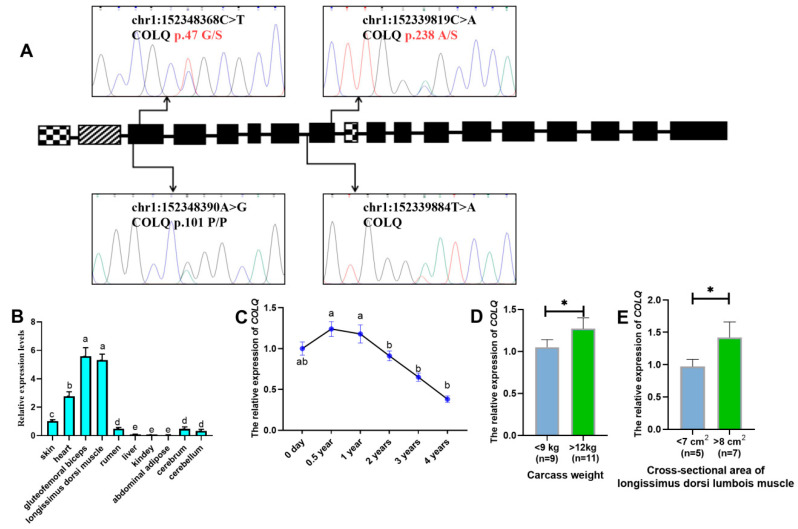
Identification of SNPs and expression of the *COLQ* gene in goats. (**A**) Localization and identification of SNPs in the *COLQ* gene by Sanger sequencing. The black module represents the exon regions shared by all three transcript variants. The mosaic module on the far left indicates the exon region unique to the COLQ-201 transcript (ENSCHIT00000016986.1). The central mosaic module denotes the exon regions common to both COLQ-201 and COLQ-202 transcripts (ENSCHIT00000016986.1; ENSCHIT00000017002.1). The hatched module shows the exon regions shared between COLQ-202 and COLQ-203 transcripts (ENSCHIT00000017002.1; ENSCHIT00000017011.1). The two missense mutations are marked in red. (**B**) Tissue expression profile of the *COLQ* gene in adult female goats. Letters (a–e) indicate significant differences (*p* < 0.05) in expression levels among tissues. (**C**) Temporal expression profile of the *COLQ* gene in longissimus dorsi muscle. Letters (a, b) indicate significant differences (*p* < 0.05) in expression levels across time points. (**D**) Expression of the *COLQ* gene in individuals with different extreme carcass weights. The * indicates significant differences (*p* < 0.05) between groups. (**E**) Expression of the *COLQ* gene in individuals with different extreme cross-sectional areas of longissimus dorsi muscle. The * indicates significant differences (*p* < 0.05) between groups.

Sanger sequencing revealed four genetic variants (Figure 1A); two were identified as missense mutations (chr1:152339819 C>A, COLQ p.238A/S; chr1:152348368 C>T, COLQ p.47G/S)**.** A mutation was identified in an intronic region (chr1:152339884 T>A, COLQ), while the other mutation (chr1:152348390 A>G, COLQ p.101P/P) was classified as synonymous mutation based on CDS alignment using UniProt and Ensembl databases. Genotype frequency distributions for the four identified SNPs are presented in Table 1. All variants displayed intermediate polymorphic information content (PIC values: 0.25–0.50), indicating moderate genetic diversity. Furthermore, strong linkage disequilibrium was observed between SNP1 (p.238A/S) and SNP2 (chr1:152339884 T>A) (Figure S1).

### 3.2. The Impact of Missense Mutations on the Structure of the COLQ Protein

The gene homology analysis revealed that the goat *COLQ* gene exhibited the closest phylogenetic relationships with those of sheep, Bovinae, Siberian musk deer, and Yarkand deer (Figure S2), which is consistent with their evolutionary divergence patterns. Structural predictions of wild-type and mutant COLQ protein tertiary structures (Figures S3 and S4) revealed conformational differences between the two variants. The magnified views highlight regions of structural divergence (denoted by black dashed circles) that may result from amino acid substitutions, insertions, or deletions. These structural variations could potentially affect protein stability, enzymatic activity, or molecular interaction properties.

### 3.3. The mRNA Expression of COLQ in LZBGs

We systematically analyzed the mRNA expression profile of *COLQ* in multiple tissues from adult LZBGs. Ubiquitous expression of *COLQ* was detected in all examined tissues (Figure 1B), with the highest expression levels observed in cardiac and skeletal muscles, particularly in the heart, gluteofemoral biceps, and longissimus dorsi. Notably, the longissimus dorsi muscle exhibited significant differential *COLQ* mRNA expression between juvenile (0.5–1 year) and sexually/physically mature (2–4 years) developmental stages (Figure 1C). Furthermore, individuals with extreme phenotypic values for the cross-sectional area of longissimus dorsi (<7 cm^2^ vs. >8 cm^2^) and carcass weight (<9 kg vs. >12 kg) demonstrated significant differences in *COLQ* expression levels (Figure 1D).

### 3.4. Association Analysis of COLQ Genetic Variants with Phenotypic Traits

Table 1 summarizes the genotype frequency distributions and population genetic parameters of the four identified genetic variants. With the exception of SNP4 (p.101P/P), all three mutations were in Hardy–Weinberg equilibrium. The polymorphism information content (PIC) analysis demonstrated that all four loci exhibited intermediate polymorphism levels, rendering them suitable as genetic markers for population genetic or association studies. Notably, SNP4 displayed the highest PIC value (0.375), indicating the greatest informativeness; however, its deviation from Hardy–Weinberg equilibrium (*p* < 0.05) suggests potential population stratification or selection pressure, warranting cautious interpretation in subsequent analyses. Genetic association analysis of goat *COLQ* polymorphisms with growth and carcass traits identified SNP1 (p.Ala238Ser) as significantly associated (*p* < 0.01) with multiple zootechnical parameters: body height, chest width, chest circumference, withers height, hip width, body weight, carcass weight, and longissimus dorsi muscle cross-sectional area (Table 2). Significant phenotypic advantages were observed in wild-type homozygotes over mutant homozygotes for seven traits (excluding body height) and over heterozygotes for six traits (excluding body weight and longissimus dorsi area). SNP2 (g.152339884T>A) showed significant correlations with body height, withers height, and body weight (Table S2). SNP3 (p.47G/S) demonstrated significant associations with body height, chest circumference, body weight, carcass weight, cross-section area of longissimus dorsi lumbois muscle, and shear force (Table 3). Significant phenotypic advantages were observed in wild-type homozygotes over mutant homozygotes for six traits and over heterozygotes for five traits (excluding body weight and longissimus dorsi area). Of particular significance, SNP4 (p.101P/P) was exclusively associated with shear force (Table S3).

### 3.5. Functional Impact of Missense Variants on COLQ Transcriptional Regulation

**Figure 2:**
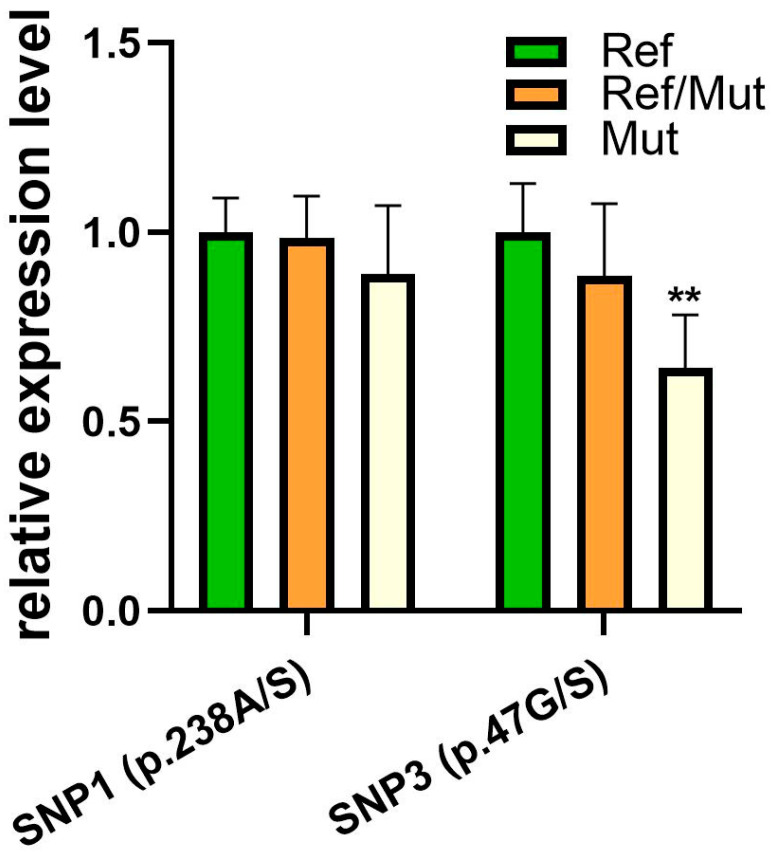
Missense mutations affect the expression of the COLQ gene in goat longissimus dorsi muscle. Comparison with normal genotype; ** indicates *p* < 0.01.

We investigated the differences in the corresponding mRNA expression levels in the longissimus dorsi muscle of female goats (Figure 2) among different SNP genotype groups. The genotype did not affect (*p* > 0.05) the *COLQ* expression in SNP1 (p.238A/S), while the *COLQ* expression in SNP3 (p.47G/S) was lower (*p* < 0.01) in the Mut genotype than in the Ref and Ref/Mut genotypes, being similar (*p* > 0.05) between the latter. Notably, the mutant homozygote of SNP3 (p.47G/S) exhibited a 0.64-fold reduction in *COLQ* expression compared to the wild-type.

## 4. Discussion

Functioning as a specialized collagen, *COLQ* serves a crucial structural role by mediating the stable attachment of acetylcholinesterase (AChE) to the synaptic basal lamina within neuromuscular junctions (NMJs) [23]. Moreover, *COLQ* participates in the long-term maintenance of neuromuscular junction (NMJ) stability through its interaction with the MuSK-mediated signaling pathway [24]. MuSK is a receptor tyrosine kinase specifically expressed on the muscle cell membrane. As a key regulator of neuromuscular junction (NMJ) formation, its activation recruits downstream proteins (such as Dok-7 and Rapsyn), mediates the clustering and stabilization of acetylcholine receptors (AChRs), and modulates the expression profile of synapse-related genes [25]. The function of *COLQ*-anchored acetylcholinesterase (AChE) depends on the integrity of the MuSK signaling pathway. Mutations in *COLQ* may disrupt this pathway, leading to impaired neuromuscular transmission and subsequently affecting muscle development and meat traits in goats [26]. Previous studies have confirmed that pathogenic mutations in the *COLQ* gene are a known cause of congenital myasthenic syndrome related to acetylcholinesterase deficiency (CMS-EAD) [27]. The phenotypic manifestations are likely linked to mechanisms of MuSK signaling deficiency that cause structural abnormalities at the NMJ and myasthenic symptoms, thereby providing a theoretical basis for explaining the phenotypes induced by *COLQ* mutations in Leizhou Black Goats [28]. In the present study, *COLQ* exhibited predominant expression in muscular tissues. Notably, significant differential expression of *COLQ* was observed in the longissimus dorsi muscle between juvenile and sexually/physically mature stages in goats (*Capra hircus*), suggesting its potential involvement in myogenesis and muscle maturation regulatory processes. Furthermore, individuals exhibiting extreme phenotypic values for carcass weight and longissimus dorsi cross-sectional area demonstrated significant variations in *COLQ* expression levels, indicating a putative dose-dependent relationship between its expression and muscular development.

Herein, we identified four SNPs, namely, two missense mutations (SNP1: p.238A/S; SNP3: p.47G/S), one synonymous mutation (SNP4: p.101P/P), and one intronic variant (SNP2: chr1:152339884 T>A). Both missense mutations were located within functional domains of the COLQ protein. Homology modeling revealed that SNP1 and SNP3 induced localized conformational changes, potentially impairing acetylcholine esterase binding capacity and thereby disrupting neuromuscular signal transduction [29,30]. Notably, SNP3, situated in a conserved N-terminal region, significantly reduced *COLQ* mRNA expression and exhibited associations with both growth and meat quality traits. This functional impact may be mediated through diminished AChE stability or via interference with *COLQ* transcriptional activity [10], suggesting its role as a key causative mutation. Although SNP1 did not markedly alter expression levels, it correlated with multiple growth traits (e.g., body height and cross-sectional area of longissimus dorsi lumbois muscle), possibly exerting effects through post-translational modifications such as protein folding or enzymatic activity [31]. The intronic mutation (SNP2) showed strong linkage disequilibrium with SNP1, implying potential synergistic regulation of *COLQ* function and highlighting haplotype effects that may surpass individual SNP contributions. The synonymous SNP4, while not altering the amino acid sequence, demonstrated an association with shear force, possibly attributable to effects on mRNA stability [32], splicing efficiency [33], or linkage disequilibrium with functional SNPs via epigenetic mechanisms [34]. In the association analysis, the wild-type homozygotes consistently exhibited optimal phenotypic values across all measured traits for each SNP, indicating that these mutations represent loss-of-function variants. In practical breeding applications, early screening and elimination of these deleterious alleles could effectively reduce their frequency within the population, thereby improving the overall phenotypic mean [35,36].

These data indicate that the SNP3 (p.47G/S) variant modulates COLQ expression and shows potential as a molecular marker for breeding goats with improved growth and muscling phenotypes. However, its functional effects require validation in larger populations. Further in vitro studies, including *COLQ* knockdown/overexpression in myocytes, are needed to elucidate its direct impact on myocyte differentiation, along with enzymatic activity assays of the mutant protein, to facilitate its application in molecular breeding programs.

## 5. Conclusions

This study identified two missense mutations (SNP1 and SNP3) in the *COLQ* gene that are significantly associated with growth and carcass traits in Leizhou Black Goats. SNP3 reduces *COLQ* expression, making it a promising marker for breeding and providing new insights into its role in muscle development.

## Figures and Tables

**Table 1:** Genotypic frequencies and population parameters in the *COLQ* gene.

Loci	Size	Genotypic Frequencies			HWE	Population Parameters			
	N	Ref	Ref/Mut	Mut	*p*-Value	Ho	He	Ne	PIC
SNP1 p.238A/S	996	0.089	0.422	0.489	*p* > 0.05	0.422	0.420	1.720	0.332
SNP2 g.152339884T>A	1002	0.076	0.423	0.501	*p* > 0.05	0.423	0.409	1.690	0.325
SNP3 p.47G/S	997	0.540	0.378	0.082	*p* > 0.05	0.378	0.395	1.650	0.317
SNP4 p.101P/P	1005	0.191	0.598	0.211	*p* > 0.05	0.598	0.500	2.000	0.375

Note: HWE, Hardy–Weinberg equilibrium. Ho, observed homozygosity. He, heterozygosity. Ne, effective allele numbers. PIC, polymorphism information content.

**Table 2:** The association analysis between the traits and SNP1 p.238A/S in the goat *COLQ* gene.

Traits	Genotypes (Mean ± SE)			*p* Values
	Ref	Ref/Mut	Mut	
body height (cm)	54.21 ^a^ ± 0.42	53.02 ^b^ ± 0.36	53.44 ^ab^ ± 0.33	0.031
chest depth (cm)	26.47 ± 0.30	26.41 ± 0.19	26.30 ± 0.23	0.341
chest width (cm)	15.83 ^a^ ± 0.17	15.68 ^b^ ± 0.23	15.64 ^b^ ± 0.19	0.026
body length (cm)	63.77 ± 0.32	63.69 ± 0.35	63.74 ± 0.41	0.842
chest circumference (cm)	71.33 ^a^ ± 0.43	69.69 ^b^ ± 0.46	70.15 ^b^ ± 0.38	0.041
withers height (cm)	56.17 ^a^ ± 0.43	54.62 ^b^ ± 0.35	54.73 ^b^ ± 0.28	0.019
hip width (cm)	17.77 ± 0.28	17.39 ± 0.19	17.51 ± 0.24	0.042
body weight (kg)	28.85 ^a^ ± 0.32	27.33 ^b^ ± 0.38	27.41 ^b^ ± 0.47	0.037
carcass weight (kg)	9.65 ^a^ ± 0.23	9.44 ^a^ ± 0.11	9.20 ^b^ ± 0.17	0.047
cross-section area of longissimus dorsi muscle (cm^2^)	7.71 ^a^ ± 0.14	7.53 ^a^ ± 0.26	7.16 ^b^ ± 0.21	0.022
water loss rate (%)	4.51 ± 0.23	4.73 ± 0.11	4.65 ± 0.14	0.181
water holding capacity (%)	4.76 ± 0.14	4.53 ± 0.17	4.58 ± 0.08	0.407
shear force (N)	48.88 ± 0.24	49.01 ± 0.28	48.32 ± 0.31	0.083

Note: letters (a, b) indicate significant differences (*p* < 0.05) between different genotypes and traits.

**Table 3:** The association analysis between the traits and SNP3 p.47G/S in the goat *COLQ* gene.

Traits	Genotypes (Mean ± SE)			*p* Values
	Ref	Ref/Mut	Mut	
body height (cm)	52.91 ^a^ ± 0.19	52.92 ^a^ ± 0.28	51.24 ^b^ ± 0.49	0.047
chest depth (cm)	26.33 ± 0.18	26.21 ± 0.19	26.31 ± 0.27	0.341
chest width (cm)	15.54 ± 0.15	15.56 ± 0.27	15.23 ± 0.33	0.268
body length (cm)	63.91 ± 0.37	62.80 ± 0.42	62.66 ± 0.42	0.427
chest circumference (cm)	70.82 ^a^ ± 0.24	69.35 ^a^ ± 0.27	67.37 ^b^ ± 0.41	0.024
withers height (cm)	54.97 ± 0.36	54.85 ± 0.24	53.42 ± 0.47	0.062
hip width (cm)	17.52 ± 0.21	17.09 ± 0.17	16.92 ± 0.11	0.207
body weight (kg)	27.88 ^a^ ± 0.25	27.07 ^a^ ± 0.32	25.96 ^b^ ± 0.21	0.021
carcass weight (kg)	9.71 ^a^ ± 0.19	9.33 ^b^ ± 0.16	9.29 ^b^ ± 0.14	0.039
cross-section area of longissimus dorsi muscle (cm^2^)	7.74 ^a^ ± 0.12	7.31 ^b^ ± 0.20	7.39 ^b^ ± 0.28	0.044
water loss rate (%)	4.61 ± 0.15	4.52 ± 0.18	4.83 ± 0.21	0.506
water holding capacity (%)	4.68 ± 0.10	4.64 ± 0.13	4.59 ± 0.09	0.072
shear force (N)	49.03 ^a^ ± 0.22	48.77 ^a^ ± 0.38	47.38 ^b^ ± 0.25	0.023

Note: letters (a, b) indicate significant differences (*p* < 0.05) between different genotypes and traits.

## Data Availability

The original contributions presented in this study are included in the article/Supplementary Materials. Further inquiries can be directed to the corresponding author.

## Supplementary Materials

The following supporting information can be downloaded at: https://www.mdpi.com/article/10.3390/ani15172618/s1. File S1: List of SNPs obtained by transcriptome sequencing annotation of LZBG. Table S1: Primer information; Table S2: Association analysis between traits and SNP2 g.152339884T>A in the goat *COLQ* gene; Table S3: Association analysis between traits and SNP4 p.101P/P in the goat *COLQ* gene; Figure S1: Linkage disequilibrium (LD) heatmap of SNPs in the goat *COLQ* gene; Figure S2: Ensembl gene trees of the *COLQ* gene; Figures S3 and S4: Predicted tertiary structures of wild-type and mutant COLQ proteins.
