# Supplementary material for: Effects of COLQ Gene Missense Mutations on Growth and Meat Traits in Leizhou Black Goats
Source: Animals (Basel). 2025 Sep 6;15(17):2618. doi: 10.3390/ani15172618 (PMC12427313; doi:10.3390/ani15172618)
Supplement: Supplementary file 1 [file animals-15-02618-s001.zip › Table S1, S2, S3.pdf]

Supplementary Tables

Table S1. Primer information

Gene name		5'-3'	Length	Note
COLQ1	F	AGCAAAAGGCGCATTGACAG	651	chr1:152339307-152339957
	R	GCCTATGGCTCTCCTGGTTC		
	F	TGATAATCCCCAGCCCAGGA	636	chr1:152339701-152340336
	R	TCTGCTTGGCCGCAAATCTA		
COLQ2	F	GAGACAGTCAGCGGTACTCA	929	chr1:152348264-152349192
	R	AGGGATCAGGATACCCGTCA		
COLQ	F	CTGCCTCCCTCTTCCTGTTC	280	mRNA-qPCR
	R	CCAGGCAGGGCCAAACA		
GADPH	F	TGAAGGTCGGTGTGAACGGATTTGG	277	mRNA-qPCR
	R	ACGACATACTCAGCACCAGCATCAC		

Note: The COLQ primer pair (amplicon size: 651 bp) was excluded from subsequent analyses due to suboptimal amplification performance as determined by agarose gel electrophoresis.

Table S2. The association analysis between the traits and SNP2 g.152339884T>A in the goat *COLQ* gene.

Traits	Genotypes（Mean ± SE）			*P* Values
	Ref	Ref/Mut	Mut	
**body height (BH, cm)**	**55.17^{a}±0.21**	**53.07^{b}±0.22**	**52.92^{b}±0.38**	0.027
Chest depth (CD, cm)	27.05±0.43	26.48±0.27	26.36±0.35	0.331
chest width (CHW, cm)	16.06±0.28	15.57±0.19	15.88±0.26	0.405
body length (BL, cm)	64.07±0.44	63.69±0.45	63.55±0.41	0.379
chest circumference (CC, cm)	71.53±0.42	69.82±0.37	70.01±0.42	0.162
**withers height (WH, cm)**	**56.35^{a}±0.32**	**55.02^{b}±0.33**	**54.63^{b}±0.48**	**0.021**
hip width (HW, cm)	17.98±0.21	17.41±0.16	17.35±0.09	0.254
**body weight (BW, kg)**	**28.35^{a}±0.41**	**27.65^{a}±0.38**	**26.43^{b}±0.20**	**0.040**

Table S3. The association analysis between the traits and SNP4 p.101P/P in the goat *COLQ* gene.

Traits	Genotypes（Mean ± SE）			*P* Values
	Ref	Ref/Mut	Mut	
body height (BH, cm)	53.04±0.41	52.79±0.36	52.91±0.30	0.882
Chest depth (CD, cm)	26.54±0.18	26.32±0.20	25.97±0.29	0.311
chest width (CHW, cm)	16.03±0.19	15.62±0.24	15.78±0.17	0.055
body length (BL, cm)	63.86±0.35	63.83±0.48	62.75±0.33	0.394
chest circumference (CC, cm)	71.16±0.31	70.38±0.47	70.43±0.39	0.058
withers height (WH, cm)	54.83±0.39	54.92±0.41	54.75±0.22	0.301
hip width (HW, cm)	17.52±0.39	17.24±0.17	17.41±0.35	0.082
body weight (BW, kg)	27.75±0.46	27.66±0.37	28.04±0.42	0.104
Carcass weight (CW, kg)	9.64±0.21	9.45±0.18	9.33±0.24	0.060
cross-section area of longissimus dorsi *lumbois*muscle (CALM, cm^2)	7.62±0.29	7.35±0.21	7.47±0.17	0.271
water loss rate (WLR, %)	4.64±0.21	4.55±0.12	4.72±0.18	0.704
water holding capacity (WHC, %)	4.69±0.09	4.53±0.14	4.60±0.23	0.266
**shear force (SF, N)**	**49.10^a±0.26**	**48.50^a±0.30**	**48.12^b±0.27**	**0.048**
