# Supplementary figures and images for: Effects of COLQ Gene Missense Mutations on Growth and Meat Traits in Leizhou Black Goats
Source: Animals (Basel). 2025 Sep 6;15(17):2618. doi: 10.3390/ani15172618 (PMC12427313; doi:10.3390/ani15172618)

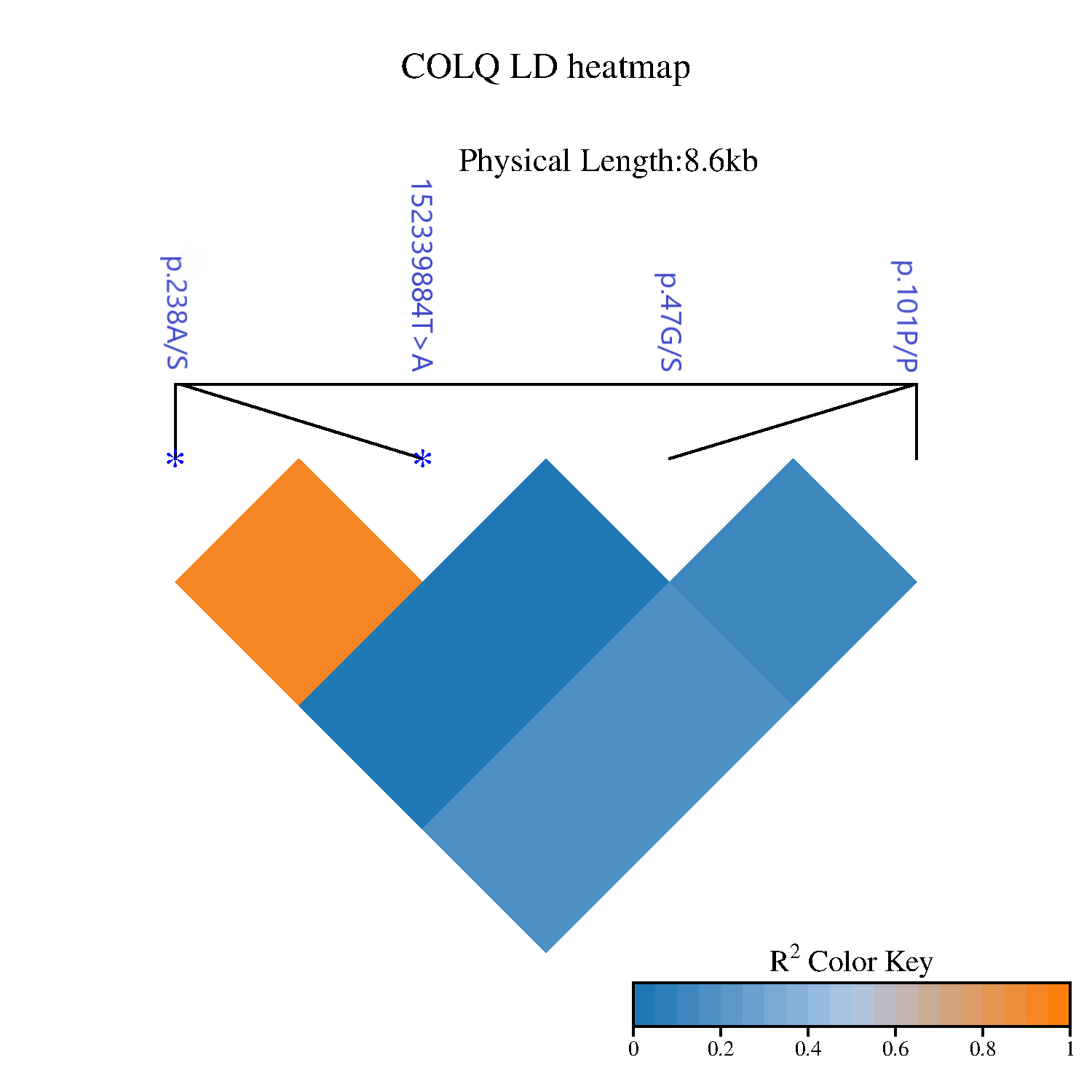

Supplement: Supplementary file 1 [file animals-15-02618-s001.zip › Figure S1.png.png]

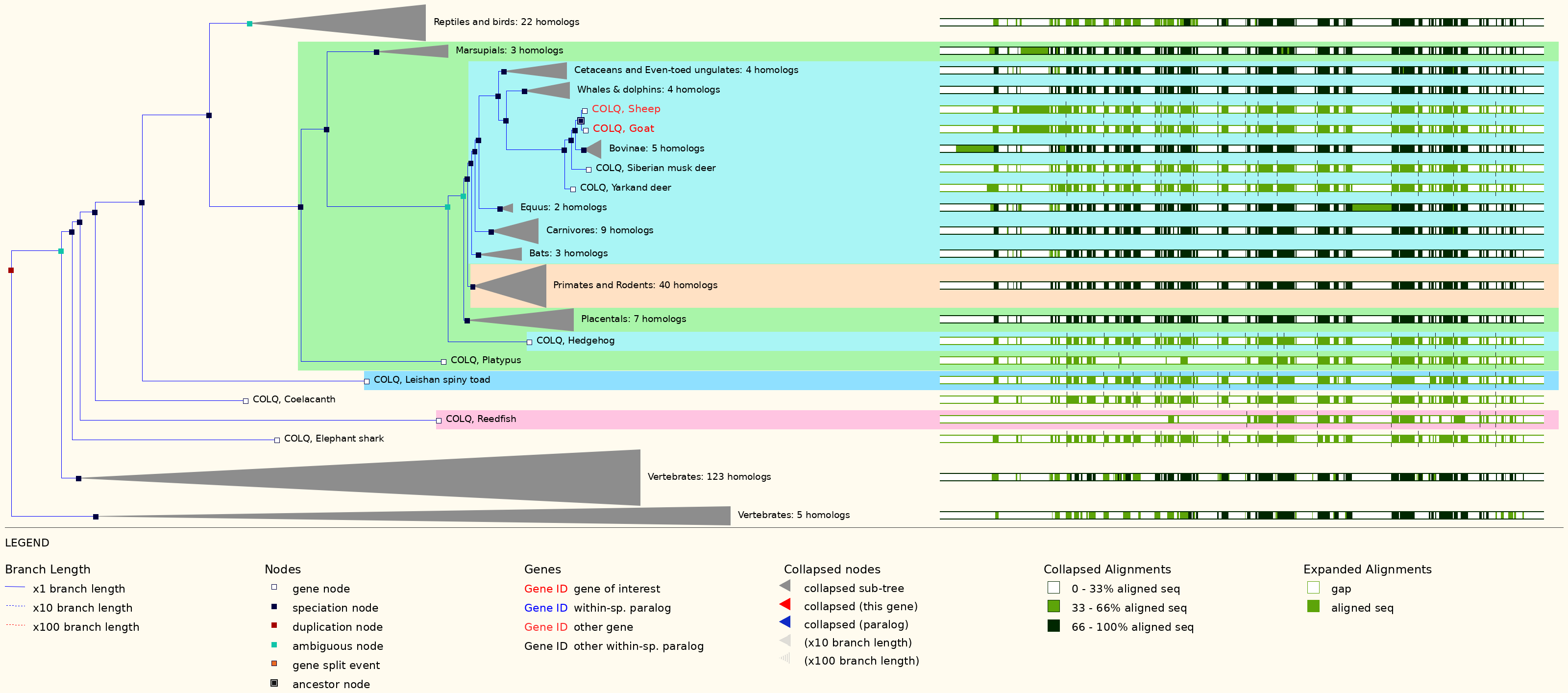

Supplement: Supplementary file 1 [file animals-15-02618-s001.zip › Figure S2.png.png]

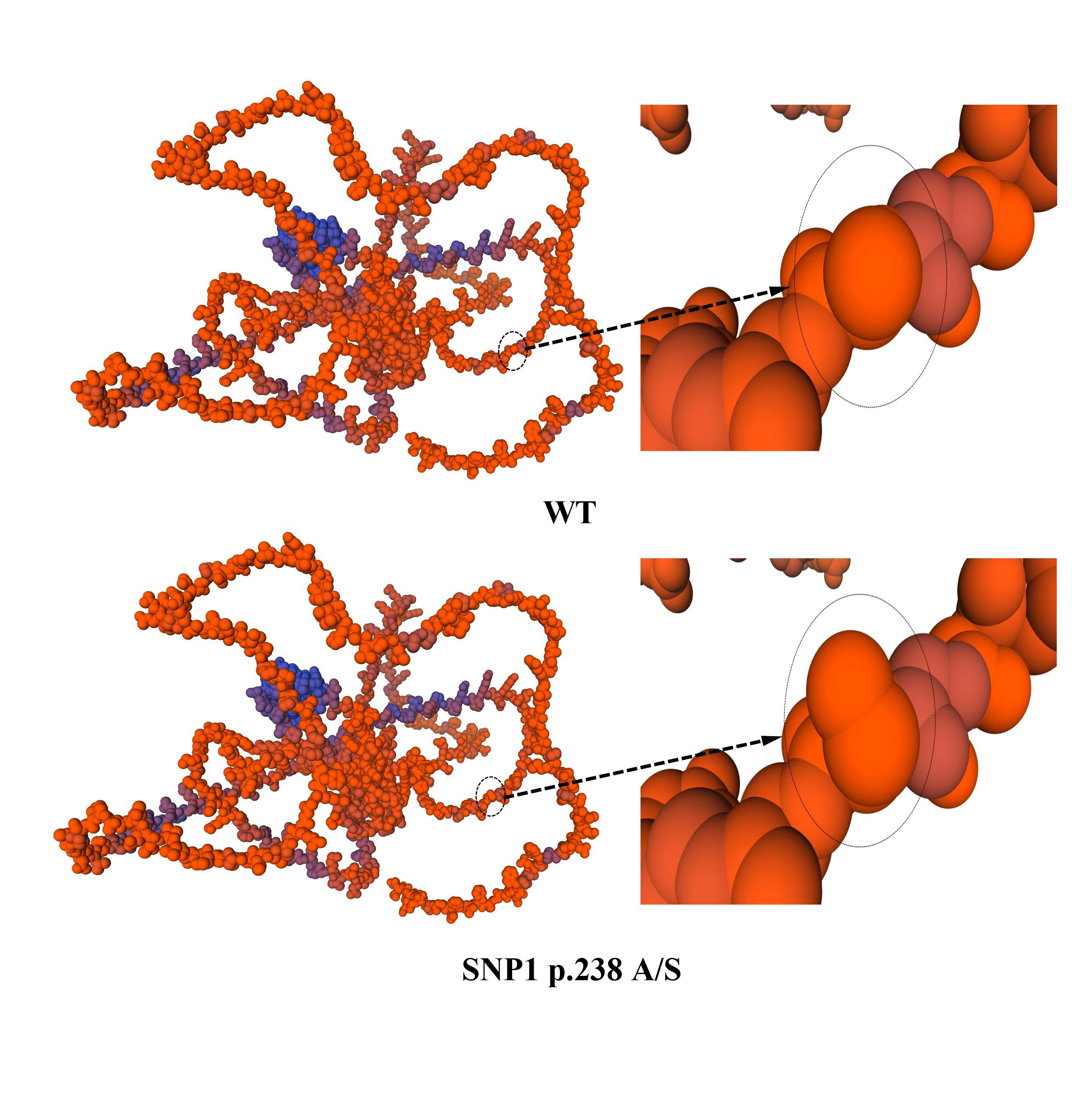

Supplement: Supplementary file 1 [file animals-15-02618-s001.zip › Figure S3..jpg]

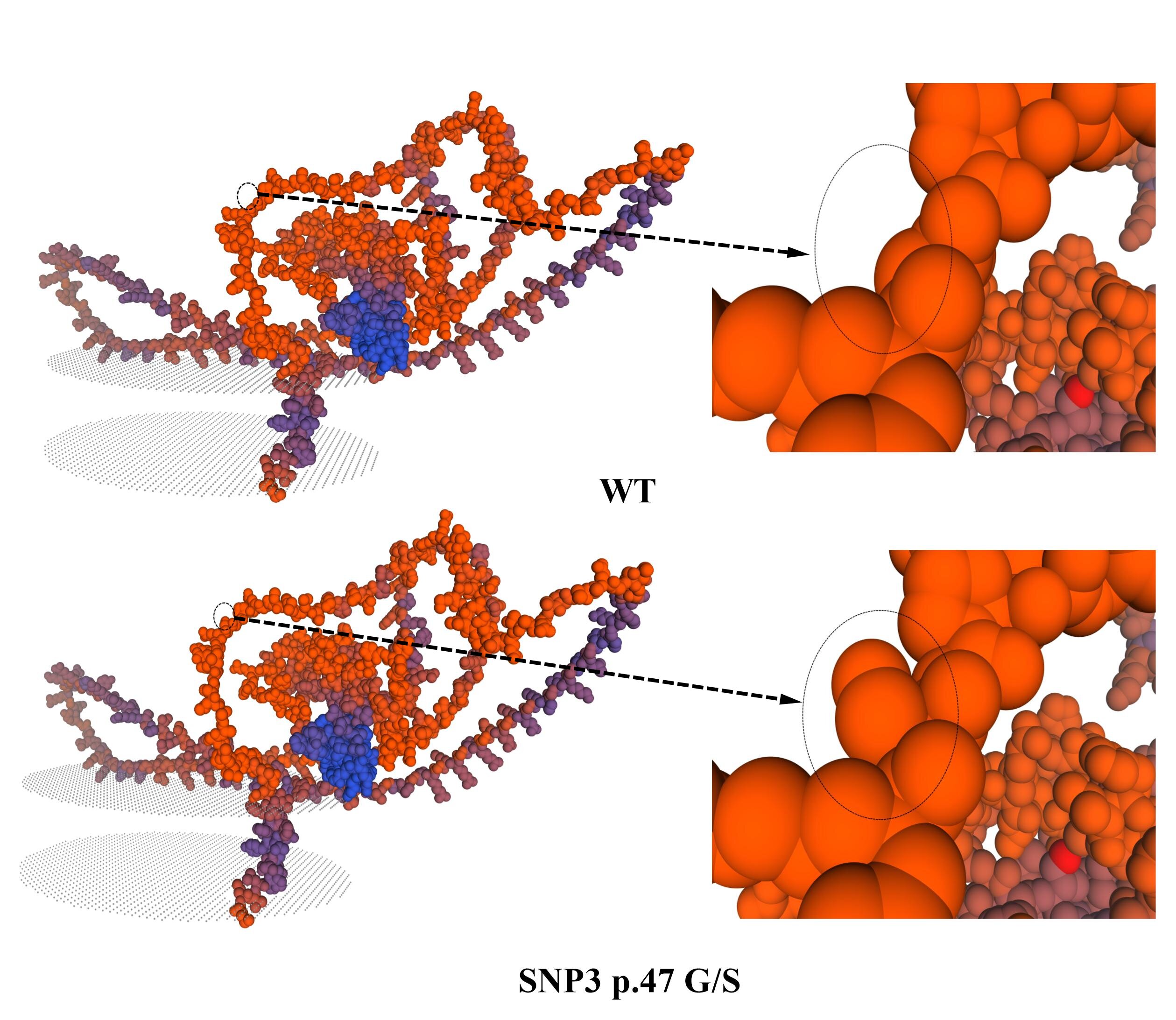

Supplement: Supplementary file 1 [file animals-15-02618-s001.zip › Figure S4..jpg]
